# Elucidating Functions of FleQ in *Xanthomonas oryzae* pv*. oryzae* by Comparative Proteomic and Phenotypic Analyses

**DOI:** 10.3390/ijms19103038

**Published:** 2018-10-05

**Authors:** Nahee Bae, Hye-Jee Park, Hanbi Park, Minyoung Kim, Eunsoo Do, Sang-Wook Han

**Affiliations:** 1 Department of Integrative Plant Science, Chung-Ang University, Anseong 17456, Korea; hi_nahee@daum.net (N.B.); hjks42@hanmail.net (H-J.P.); feelings57@naver.com (H.P.); minyoung0622@naver.com (M.K.); 2 Department of Systems Biotechnology, Chung-Ang University, Anseong 17456, Korea; powerjin19@cau.ac.kr

**Keywords:** comparative proteomics, *Xanthomonas*, FleQ, σ^54^-dependent transcription activator

## Abstract

To acclimate to different environments, gene expression has to be controlled using diverse transcriptional activators. FleQ activates σ^54^-dependent transcription initiation and regulates flagellar biosynthesis and other mechanisms in several bacteria. *Xanthomonas oryzae* pv. *oryzae* (*Xoo*), which is a causal agent of bacterial leaf blight on rice, lacking FleQ loses swimming motility and virulence is not altered. However, other biological mechanisms related with FleQ in *Xoo* are unknown. In this study, we generated the FleQ-overexpressing strain, *Xoo*(FleQ), and knockout mutant, *XooΔfleQ*. To predict the mechanisms affected by FleQ, label-free shotgun comparative proteomics was carried out. Based on proteomic results, we performed diverse phenotypic assays. *Xoo*(FleQ) had reduced ability to elicit disease symptoms and exopolysaccharide production. Additionally, the ability of *XooΔfleQ*(EV) (empty vector) and *Xoo*(FleQ) to form biofilm was decreased. Swarming motility of *XooΔfleQ*(EV) was abolished, but was only reduced for *Xoo*(FleQ). Additionally, abnormal twitching motility was observed in both strains. Siderophore production of *Xoo*(FleQ) was enhanced in iron-rich conditions. The proteomic and phenotypic analyses revealed that FleQ is involved in flagellar-dependent motility and other mechanisms, including symptom development, twitching motility, exopolysaccharide production, biofilm formation, and siderophore production. Thus, this study provides fundamental information about a σ^54^-dependent transcription activator in *Xoo*.

## 1. Introduction

Bacterial leaf blight disease caused by *Xanthomonas oryzae* pv. *oryzae* (*Xoo*) is one of the most destructive plant diseases and leads to rice yield losses up to 50% in countries where rice has been cultivated as a staple crop [1]. *Xoo* enters leaves through wounds or hydathodes, and spreads in the xylem [2]. White or gray wilt symptoms appear on diseased plants after infection. *Xoo* produces diverse virulence factors such as exopolysaccharide (EPS), biofilm, extracellular enzymes, and type III effectors [3,4,5]. *Xoo* is regarded as an important model bacterium to investigate the molecular mechanisms of plant–bacteria pathogenesis.

To adapt to environments and cause disease, bacteria regulate their gene expression. RNA polymerases interact with sigma (σ) factors to initiate transcription, and the sigma factors cooperate with transcription activators [6]. These transcription activators cooperating with a sigma factor control gene expression to regulate biological behaviors. For example, PhoB, which is a transcriptional activator with a σ^70^ factor, controls regulatory networks under phosphate starvation in *Escherichia coli* and WhiB7, which activates transcription by interacting with sigma factor SigA, is important for resistance to several antibiotics [7,8]. FleQ is known as a σ^54^-dependent transcription activator in *Pseudomonas aeruginosa*, *X. campestris* pv. *campestris* (*Xcc*), and *Xoo* [9,10,11].

The participation of FleQ as σ-^54^dependent transcriptional activator has been well-studied in *Pseudomonas* spp. In *P. aeruginosa*, FleQ is involved in flagellar-dependent motility, mucin adhesion, and expression of biofilm-related genes [12,13]. Transcriptome analysis in *P. putida* showed that FleQ was related to not only flagellar assembly, but also the type VI secretion system, cyclic diguanylate (c-di-GMP) biosynthesis, and cellulose biosynthesis [14]. In addition, RNA arbitrarily primed polymerase chain reaction revealed that FleQ is involved in regulating the expression of genes encoding topoisomerase, halovibrin A, phosphoglycerate kinase, and potassium efflux proteins in *Vibrio fischeri* [15]. In *Xoo*, a *fleQ*-knockout mutant displayed abolished swimming motility but virulence was not altered on susceptible rice plants [10]. However, other cellular and biological mechanisms controlled by FleQ in *Xoo* remain unknown.

In this study, we investigated the functions of FleQ (Locus Tag: PXO_00993; Accession Number: ACD59169) by creating a *fleQ*-knockout mutant, *XooΔfleQ*, and a FleQ-overexpressing strain, *Xoo*(FleQ). A label-free comparative shotgun proteomic analysis and clusters of orthologous groups (COGs) were carried out to assess the biological mechanisms associated with FleQ. Based on proteomic results, diverse phenotypic assays were carried out. The participation of FleQ in *Xoo* virulence and two types of motility were identified, together with its role in extracellular polysaccharide (EPS) production, biofilm formation, and siderophore production.

## 2. Results

### 2.1. FleQ Is Involved in Controlling Expression of Genes Encoding Diverse Proteins

**Figure 1:**
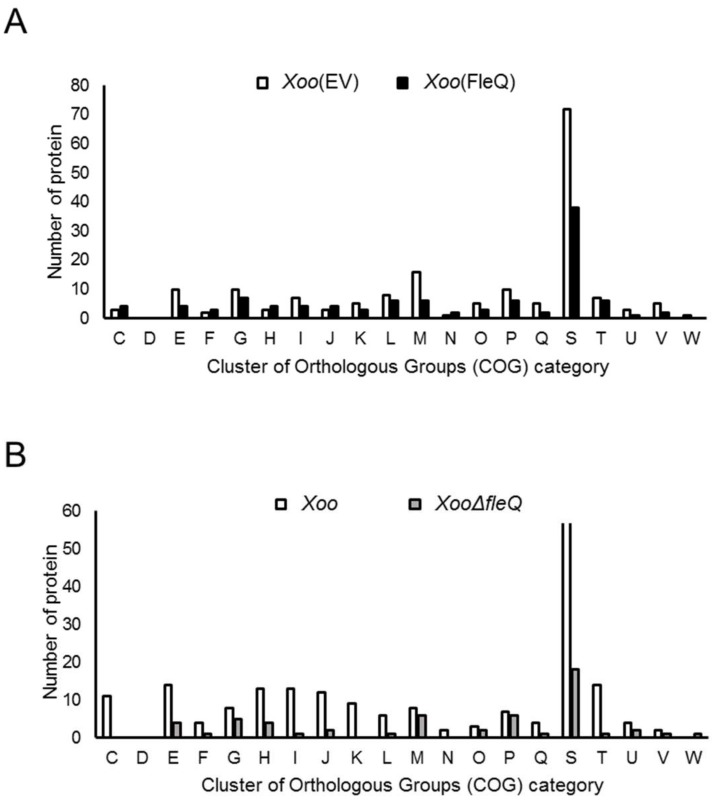
Comparison of the abundance of types of proteins controlled by FleQ in cluster of orthologous groups (COG) groups between (**A**) *Xanthomonas oryzae* pv. *oryzae*, empty vector (*Xoo*(EV)) vs. *Xoo*(FleQ) and (**B**) *Xoo* vs. *XooΔfleQ*. Abbreviations: C, Energy production and conversion; D, Cell cycle control and mitosis; E, Amino acid metabolism and transport; F, Nucleotide metabolism and transport; G, Carbohydrate metabolism and transport; H, Coenzyme metabolism; I, Lipid metabolism; J, Translation; K, Transcription; L, Replication and repair; M, Cell wall/membrane/envelop biogenesis; N, Cell motility; O, Post-translational modification, protein turnover, chaperone functions; P, Inorganic ion transport and metabolism; Q, Secondary structure; R, General functional prediction only; S, Function unknown; T, Signal transduction; U, Intracellular trafficking and secretion; V, Defense mechanisms.

Although FleQ has been identified as the transcription activator of flagellar synthesis in *Xoo* [10]*,* other functions related to FleQ are poorly understood and a proteomic analysis for FleQ has not been carried out. To predict biological and cellular mechanisms associated with FleQ, label-free shotgun comparative proteomic analyses were carried out from two sets of strains: X*oo*(EV) (empty vector) vs. *Xoo*(FleQ) and *Xoo* vs. *XooΔfleQ*. Numbers of detected proteins and peptide spectra matches (PSMs) in biological replicates from LC-MS/MS analysis were shown in Tables S1 and S2. The number of shared proteins in three replicates detected by LC-MS/MS were 1291 and 1244 proteins in *Xoo*(EV) and *Xoo*(FleQ), respectively, and the abundance of these proteins was compared. A total of 174 and 105 proteins were more abundant (>2-fold) in *Xoo*(EV) and *Xoo*(FleQ), respectively (Tables S3 and S4). Using COG analysis, these abundant proteins were categorized. Among the 20 groups, the number of proteins in 15 groups were higher in *Xoo*(FleQ) than *Xoo*(FleQ) (Figure 1A). Proteins belonging group M (cell wall/membrane/envelope biogenesis) are more abundant than other groups, except group S (function unknown). Interestingly, eight type III effectors (T3Es) (XopF1, XopW, XopN, Hpa3, XopQ, pthoXo1, XopK, XopL), which are modulators of host defense mechanisms, and eight Gum proteins (GumB, GumD, GumH, GumI, GumJ, GumL,GumM, GumN) related to EPS production were more abundant in *Xoo*(EV) compared with *Xoo*(FleQ) (Table S3). In addition, ferric uptake regulator (Fur) was detected in *Xoo*(FleQ), but not in *Xoo*(EV), postulating that iron uptake may be affected by overexpression of FleQ. The FleQ protein in *Xoo*(FleQ) is more abundant (55.73-fold), indicating *Xoo*(FleQ) is overexpressing FleQ proteins compared with *Xoo*(EV). 

Between *Xoo* and *XooΔfleQ*, 1227 and 1161 proteins were shared in the three replicates of *Xoo* and *XooΔfleQ*, respectively (Table S2), and the abundance of these proteins was compared. A total of 190 and 56 proteins were more abundant in *Xoo* and *XooΔfleQ,* respectively (Tables S5 and S6). Proteins in 19 groups were predominant in *Xoo*, excluding W group (Figure 1B) and the number of proteins of *Xoo* in these categories is up to 4-fold higher than that of *XooΔfleQ*. Although each group possessed fewer than six proteins in *XooΔfleQ*, group M (cell wall/membrane/envelope biogenesis) and group P (inorganic ion transport and metabolism) contained more than six proteins (Figure 1B). In addition, group K (transcription) was not present in *XooΔfleQ*. In contrast to the set, *Xoo*(EV) vs. *Xoo*(FleQ), the abundance of only two T3Es, AvrBs2 and Hpa1, was changed in *Xoo* vs. *XooΔfleQ* (Table S5 and S6). More importantly, group N (cell motility), including a flagellar protein, was not detected in *XooΔfleQ*, while it was observed in *Xoo* (Figure 1B).

### 2.2. Overexpression of FleQ Reduced Symptom Development in Rice Plants

**Figure 2:**
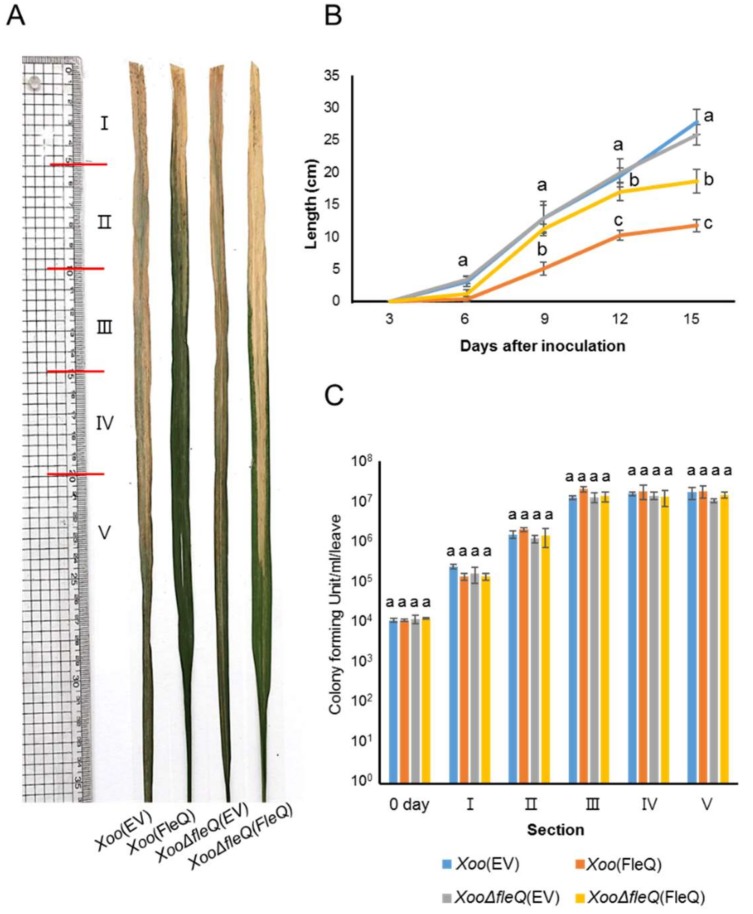
Virulence assay of *Xoo*(EV), *Xoo*(FleQ), *XooΔfleQ*(EV) and *XooΔfleQ*(FleQ) strains on rice plants. (**A**) Representative photograph of leaves taken at 15 days after inoculation (DAI). (**B**) Graph represents lesion length which was measured at 3, 6, 9, 12, and 15 DAI. (**C**) Bacterial populations were calculated at day 0 from whole leaves and 15 DAI from sections. Diseased leaves were divided into 5 cm sections up to 20 cm, and above 20 cm to the end. Bars indicate the mean of at least six leaves with the standard deviations. Different letters on the bars indicate statistically significant differences in average values by one-way ANOVA (*p* < 0.05).

Virulence in the *fleQ*-knockout mutant is not altered in *Xoo* [10]. Therefore, we tested whether the overexpression of the *fleQ* gene was involved in the virulence of *Xoo*. We inoculated four strains, *Xoo*(EV), *Xoo*(FleQ), *XooΔfleQ*(EV), and *XooΔfleQ*(FleQ), into susceptible hosts (Figure 2A) and measured lesion length at 3, 6, 9, 12, and 15 days after inoculation (DAI) (Figure 2B). Interestingly, *Xoo*(FleQ) displayed a reduced lesion length during the entire observation period, while *XooΔfleQ*(EV) did not. *XooΔfleQ*(FleQ) displayed slightly decreased lesion length in rice plants compared to *Xoo*(EV) due to copy number of the vector used for complementation. The bacterial population of the four strains was not significantly different in all sections at 15 DAI (Figure 2C). Additionally, there was no significant difference in the growth of the four strains in peptone sucrose broth (PSB) (Figure S1), indicating that FleQ is not involved in bacterial multiplication in the given condition. These results indicated that overexpression of FleQ decreased symptom development, although FleQ did not affect *Xoo* migration and multiplication in rice.

### 2.3. FleQ Affects EPS Production and Biofilm Formation

**Figure 3:**
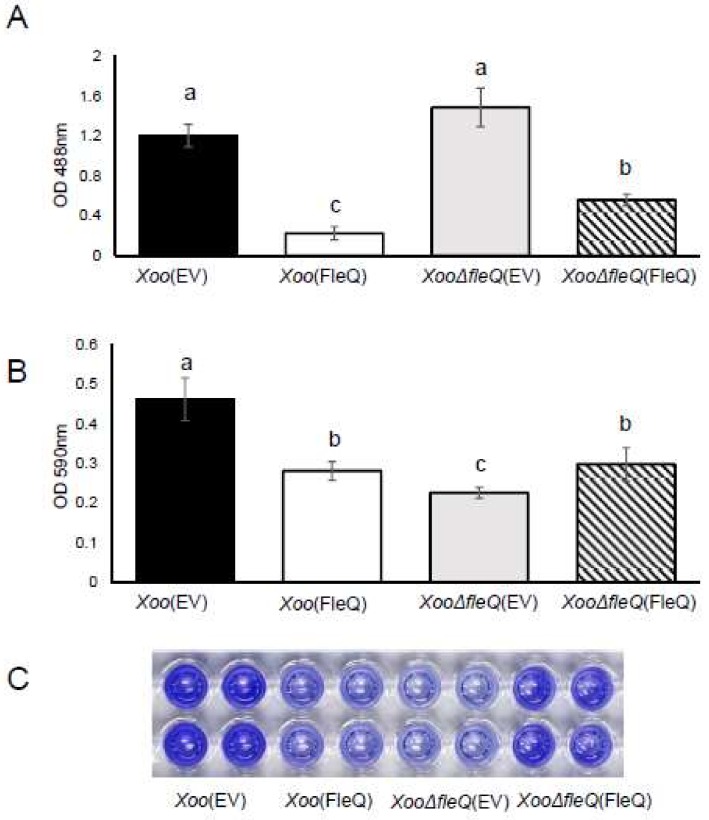
Extracellular polysaccharide (EPS) production and biofilm formation in *Xoo* strains. (**A**) After *Xoo* strains were cultured in peptone sucrose broth for 1 day and in XOM2 for 4 days, EPS was quantified by a phenol-sulfuric acid method at 488 nm. Bars represent the mean of five biological replicates with the standard deviation. (**B**) Biofilm formation from *Xoo* strains was determined by the PVC plate assay. Bars indicate the means of 12 biological replicates with the standard deviation. Different letters on the bars indicate statistically significant differences in average values by one-way ANOVA (*p* < 0.05). (**C**) The photograph of stained cells resolved in 95% EtOH solution was taken at 7 days after incubation in XOM2. OD = optical density.

Proteomic results indicated Gum proteins related with EPS biosynthesis were more prevalent in *Xoo*(EV) than *Xoo*(FleQ). Thus, EPS production was tested. EPS production in *Xoo*(FleQ) was six-fold lower than that in *Xoo*(EV). In the case of *XooΔfleQ*(EV)*,* no remarkable difference was shown compared with *Xoo*(EV) (Figure 3A). A previous study reported that EPS production is closely associated with biofilm formation and FleQ in *P. aeruginosa* is involved in biofilm formation [13]. Thus, we investigated biofilm formation (Figure 3B,C). Biofilm formation by *Xoo*(FleQ) was 1.6-fold decreased compared with *Xoo*(EV), and *XooΔfleQ*(EV) displayed the least biofilm formation ability among the four strains. The biofilm formation ability of the complemented strain was partially restored. The results suggested that FleQ is associated with EPS production and biofilm formation in *Xoo*.

### 2.4. FleQ Is Crucial for Twitching and Swarming Motility

**Figure 4:**
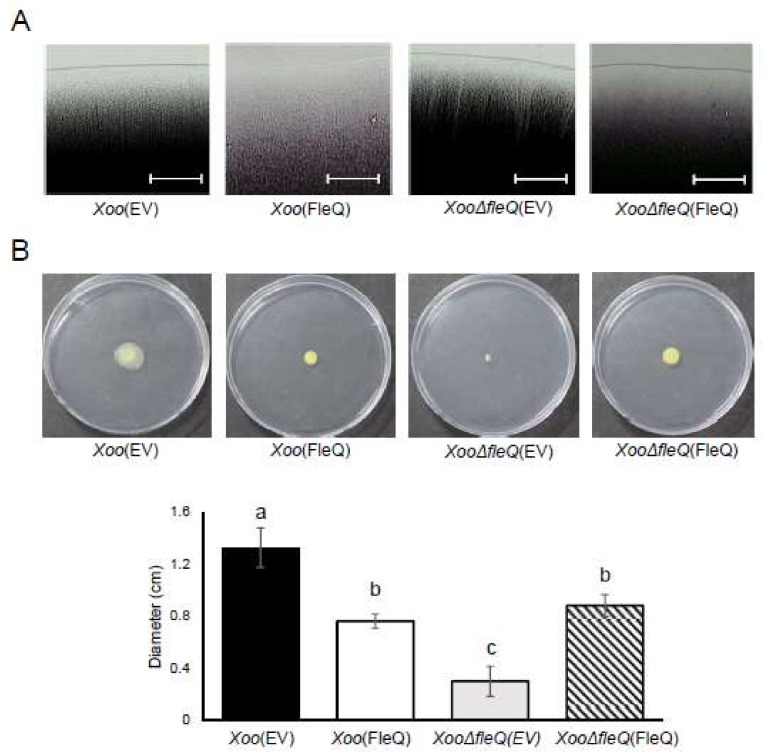
Twitching and swarming motility in *Xoo* strains. (**A**) After a 3-day incubation on a peptone sucrose agar (PSA) plate, twitching motility from the colonies was oberved by light microscopy. White bars represent 100 μm. (**B**) Swarming motility was observed by a light microscope and analyzed by measuring the diameter of colonies after 7 days incubation on semi-solid XOM2 media. Different letters on the bars indicate statistically significant differences in average values by one-way ANOVA (*p* < 0.05).

A previous study described the loss of swimming ability by a *fleQ*-knockout mutant in *Xoo* [10]. Furthermore, group N (cell motility) was not detected in *XooΔfleQ*(EV) in the comparative proteomic results (Figure 1B). To verify if FleQ is required for *Xoo* motility, swarming and twitching motility were examined. Concerning twitching motility, *Xoo*(EV) showed slender and vertical lines and appeared to have a flat edge. By contrast, *Xoo*(FleQ) and *XooΔfleQ*(FleQ) had no visible lines and displayed an uneven edge (Figure 4A). *XooΔfleQ*(EV) had thicker, imbalanced lines and a rough edge (Figure 4A). As expected, the swarming motility ability of *XooΔfleQ*(EV) was abolished and *Xoo*(FleQ) also exhibited reduced ability compared with *Xoo*(EV) (Figure 4B). The motility of the complemented strain was partially restored towards the level of the wildtype. These results clearly indicated that FleQ is involved in flagellar-dependent and flagellar-independent motility in *Xoo*.

### 2.5. FleQ Is Involved in Siderophore Production 

**Figure 5:**
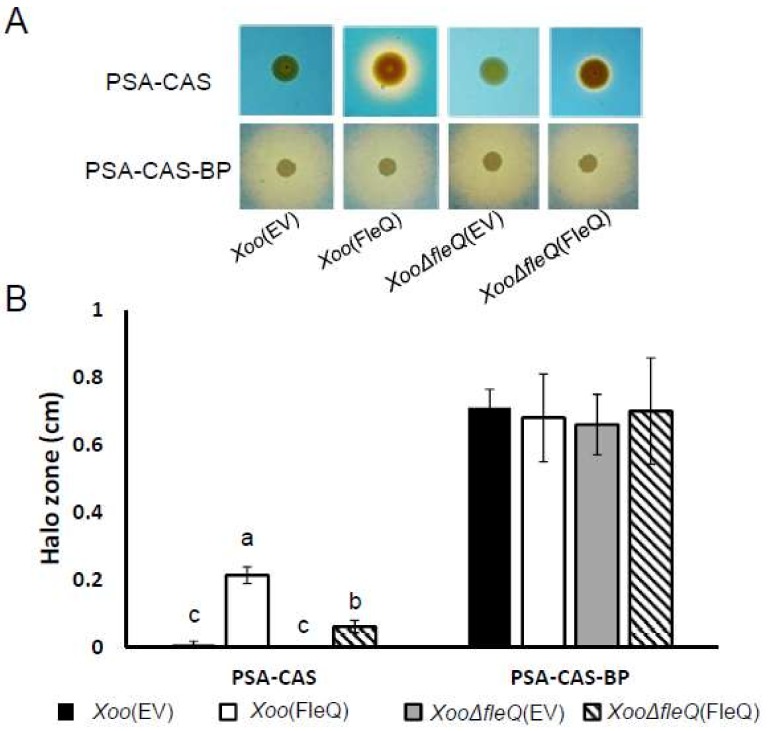
Chromeazurol S (CAS) assay in four strains, *Xoo*(EV), *Xoo*(FleQ), *XooΔfleQ*(EV) and *XooΔfleQ*(FleQ). (**A**) To show colonies on peptone sucrose agar-chromeazurol S (PSA-CAS) and PSA-CAS-BP, which contains 2,2′-dipyridyl as an iron chelator, images were taken after 7 days of incubation. (**B**) Diameter of comparative halo zones were measuered and means of halo width in each of the four strains was represented by bar graph. Different letters on the bars indicate statistically significant differences in average values by one-way ANOVA (*p* < 0.05).

Interestingly, the proteomic analysis revealed alterations in the abundance of 18 TonB-dependent receptors, which are membrane-bound and related to the uptake of large substrates like iron-siderophore complexes. Therefore, we investigated the production of the iron-chelating siderophore using the chromeazurol S (CAS) assay. In iron-rich conditions, *Xoo*(FleQ) produced a 28-fold higher level of siderophores, represented by yellow halos, than *Xoo*(EV) (Figure 5A). This increase was not observed for *XooΔfleQ*(EV). However, when the Fe^2+^ chelator 2,2′-dipyridyl was added, all strains showed similar siderophore production (Figure 5B). These data indicated that overexpression of FleQ is involved in the facilitation of siderophore production in an iron-rich environment.

## 3. Discussion

FleQ is an important factor for flagellar biosynthesis in several bacteria, including *Xoo*. Although FleQ can affect the expression of flagellar genes [10], there is no evidence of a relationship between FleQ and other mechanisms in *Xoo*. In this study, we demonstrated functions of FleQ in other biological mechanisms by comparative proteomics and phenotypic observation. In our proteomic analysis, the abundance of eight T3Es and three T3SS proteins was affected by FleQ. Among the T3Es and T3SS, XopQ, XopN, and HrpD6 are important for symptom development in host plants in *Xoo* [16,17,18]. A decrease in the level of protein abundance for T3Es and T3SS may cause reduced symptoms on rice leaves in *Xoo*(FleQ). Our proteomic analysis also showed that eight Gum proteins, including GumD and GumG, were only found in *Xoo*(EV), and were not present in *Xoo*(FleQ) (Tables S3 and S4). These proteins are crucial for EPS biosynthesis, which contributes to virulence [19]. Consequently, the ability to produce EPS in *Xoo*(FleQ) was lower than in *Xoo*(EV). The reduced EPS production in *Xoo*(FleQ) may decrease symptom development. Moreover, *Xoo* mainly colonizes in the xylem, and blocking xylem cells by EPS produced from *Xoo* is one of main factors for symptom development [2]. Because *Xoo*(FleQ) displayed reduced expression of Gum proteins and decreased EPS production, *Xoo*(FleQ) may be unable to obstruct water transfer and the development of disease in rice. 

EPS production and biofilm formation is affected by FleQ with c-di GMP in *P*. *aeruginosa* [20] and EPS is one of major constituents of biofilms in most bacteria [21]. In agreement with the observations, we demonstrated that the abilities of EPS production and biofilm formation in *Xoo*(FleQ) were reduced compared with *Xoo*(EV). However, biofilm formation in *XooΔfleQ*(EV) was not correlated with EPS production. In several vascular pathogens, including *Pantoea stewartii*, *Ralstonia solanacearum*, and *Dickeya dadantii*, flagella are involved in biofilm formation [22,23,24]. In addition, flagella are also important for mature biofilm formation in *X. axonopodis* pv. *citri* (*Xac*) [25]. *Xac* lacking flagella contain fewer water channels, which enable nutrient flow to bacterial cells in the biofilm architecture, and thin biofilm structures were observed. Our proteomic analysis revealed that *Xoo*(EV) only showed flagellar proteins compared with *XooΔfleQ*(EV) (Tables S3 and S4) and that swarming motility was abolished in the *XooΔfleQ*(EV) mutant. Therefore, it can be postulated that low biofilm formation of *XooΔfleQ*(EV) is caused by lack of flagellum in the mutant. 

Twitching motility is a flagellar-independent motility essential for host colonization and formation of biofilms in some bacteria [26]. Type IV pili (T4P), which mediate twitching motility and pili retraction, are thought to be the motive force for twitching motility [27]. Although flagellar proteins were not detected in *XooΔfleQ* (Table S5), a PilW protein, one of the T4P proteins, was found in *XooΔfleQ* in our proteomic results (Table S6). The T4P protein, FimC, was found on *Xoo*(EV), but not in *Xoo*(FleQ) (Tables S3 and S4). Additionally, abnormal twitching motility was observed in *Xoo*(FleQ) and *XooΔfleQ*(EV) (Figure 4A), which is consistent with proteomic results. FleQ is important for *Xoo* swimming motility, which is influenced by rotation of unipolar flagella, and *Xcc* flagellar synthesis [10,11]. In an agreement with these studies, we also showed that swarming motility of *XooΔfleQ*(EV) was abolished. Thus, proteomic and phenotypic analyses indicate that FleQ is involved in flagellar-dependent and independent motility. 

Iron is an essential element for survival and bacteria employ siderophores to efficiently uptake Fe^3+^ from outside sources via transport involving TonB-dependent receptors [28]. In *Xoo*, the strain lacking RpfF displayed overproduction of siderophores and were less virulent than the wildtype [29]. Similarly, *Xoo*(FleQ) increased siderophore production in an iron-rich environment and had reduced ability to show disease symptoms. Additionally, proteomic results reveal that FleQ could be associated with regulating expression of some TonB-dependent proteins. Thus, it can be speculated that the increased siderophore production reduced disease symptoms. These results support the proposition that iron metabolism contributes to the virulence of *Xanthomonas* spp.

In conclusion, we have postulated biological mechanisms related to FleQ by a label-free shotgun, comparative proteomic analysis in *Xoo*. We further showed that FleQ is associated with symptom development, EPS production, biofilm formation, motility, and siderophore production by phenotypic observation. Thus, FleQ is involved in the regulation of not only flagellar biosynthesis, but also other biological mechanisms in *Xoo*. This study will provide new insight into understanding the functions of FleQ.

## 4. Materials and Methods

### 4.1. Bacterial Strains and Growth Condition 

For this study, *X. oryzae* pv. oryzae (*Xoo*) strain PXO99A was used as the wildtype; its genome was completely sequenced [30]. *Xoo* strains were cultured in peptone sucrose medium (peptone: 10 g/L, sucrose: 10 g/L, l-glutamic acids: 1 g/L) and XOM2 medium (670 μM dl-methionine, 10 mM sodium l-glutamate, 14.7 mM KH_2_PO_4_, 40 μM MnSO_4_, 240 μM Fe(III)-EDTA, 0.18% d-xylose, and 5 mM MgCl_2_, pH 6.5) at 28 °C. *Escherichia coli* strain DH5α was grown in Lysogeny broth medium [31]. Four antibiotics were used: gentamycin (10 μg/mL), kanamycin (50 μg/mL), cephalexin (30 μg/mL), and ampicillin (100 μg/mL).

### 4.2. Generation of Xoo Strains

All plasmids and bacterial strains used in this study are listed in Table S7. To generate the insertional mutant, *XooΔfleQ*, the DNA fragment containing *fleQ* was amplified by gene specific primers (5′-atgatgccgagcatcgtgaa-3′ and 5′-gatttcgcctcgaccatcct-5′). The amplified product was cloned into the pGEM-T-easy vector and a kanamycin cassette from pUC4k was inserted in the middle of the *fleQ* gene digested by *Kpn*I. The construct was introduced into *Xoo* by electroporation. *XooΔfleQ* was selected on PSA medium containing kanamycin, but not ampicillin. To create the *fleQ*-overexpressing strain, *Xoo*(FleQ), the open reading frame of *fleQ* was amplified with the gene specific primers (5′-ctcgagatgagtgagtcccgcattctgt-3′ and 5′-aagcttcagtggtggtggtggtggtggttggccagctcggtctgctcg-3′). The amplified DNA was cloned into a pGEM-T-easy vector. The identified DNA fragment was subcloned again into a pBBR1MCS-5 vector [32] and the expression of FleQ was driven by the Lac promoter in the vector, creating pBBR1FleQ. The construct was introduced into *Xoo* and *XooΔfleQ* to generate *Xoo*(FleQ) and the complemented strain, *XooΔfleQ*(FleQ), respectively. The pBBR1MCS-5 was transferred into *Xoo* and *XooΔfleQ*, creating *Xoo*(EV) and *XooΔfleQ*(EV).

### 4.3. Label-Free Shotgun Proteomic Analysis

Incubation and harvest of *Xoo*(FleQ) and *XooΔfleQ* strains, protein extraction, peptide preparation, liquid chromatography-tandem mass spectrometry (LC-MS/MS), peptide identification, quantification, and statistical analysis were performed as described previously [33]. Briefly, *Xoo* strains were incubated in PSB, washed, adjusted to an OD_600_ of 0.6 in XOM2 medium, and then grown again for 16 h. After harvesting bacterial cells, total proteins were extracted and trypsin was used for the digestion. Tryptic-digested peptides (2 μg) of three biological replicates were analyzed using split-free nano-liquid chromatography (LC, EASY-nLC II; Thermo Fisher Scientific, Waltham, MA, USA) linked to the LTQ Velos Pro instrument (Thermo Fisher Scientific) equipped with Thermo Proteome Discoverer 1.3 software (ver. 1.3.0.399). A column packed in-house with 7.5 cm of MAGIG C18AQ 200A (5 μm) material (Michrom BioResources, Auburn, CA, USA) was used for separation. Six data-dependent MS/MS scans were used to acquire full mass spectrometry spectra. Dynamic exclusion was allowed with one repeat count, 0.5 min repeat duration, and exclusion duration of 3 min, with charge state selection permitted to take 2^+^ and 3^+^ ions by priority. The six most intense ions in each full MS scan were assembled for fragmentation and explored in centroid mode within the linear ion trap. SEQUEST software was used to analyze the obtained MS/MS spectra. The *Xoo* strain PXO99A database was used to search spectra. With opposite database searches, all collected peptides had a false discovery rate (FDR) of 0.01 and precursor mass exactness of 100 ppm. The probability score for all peptides was >20. The least two unique peptides were matched with proteins which were regarded as present in the sample. The obtained data were imported into Scaffold 4 (Proteome Software, Portland, OR, USA). Identified proteins and PSMs for *Xoo*(EV) and *Xoo* were used in a study published recently [34] and in this study for the comparison. Peptide spectra matches (PSMs) of each protein were normalized from total PSMs. The average number of normalized PSMs in three biological replicates was calculated per protein and used as a comparison value to identify differently (over two-fold) abundant proteins in two sets, *Xoo* vs. *XooΔfleQ* and *Xoo*(EV) vs. *Xoo*(FleQ). To postulate the mechanisms, clusters of orthologous groups (COG) classification were employed.

### 4.4. Virulence Test

*Oryza sativa* L. (cultivar kitaake (Kit)) plants [35] were grown in a greenhouse at 25–32 °C for 5 weeks. *Xoo* strains were grown in PSA and adjusted to an OD_600_ of 0.6. A leaf clipping method was performed. After inoculation, lesion length was checked on the infected leaves at 3, 6, 9, 12, and 15 days after inoculation. Infected leaves were divided into five groups: I (0–5), II (6–10), III (11–15), IV (16–20), and V (>21 cm). Each section was sliced into small pieces and immersed in 10 mM MgCl_2_. The extracted bacterial suspensions were serially diluted and dropped onto PSA containing appropriate antibiotics to enumerate the bacterial population. The bacterial population was assessed by colony counting. The assay was repeated at least four times with six biological replicates.

### 4.5. Motility Assay

Twitching motility was performed as described previously [36]. Briefly, *Xoo* strains grown for 2 days were adjusted to an OD_600_ of 0.6 and tested on PSA media. After 3 μL bacterial resuspension was dotted onto plates, bacteria were incubated for 3 days and observed using a light microscope (Olympus SZX10, Tokyo, Japan). This experiment was performed at least four times with four biological replicates. Swarming motility was conducted as previously described [37]. Swarming motility was investigated on media containing 0.3% agar. Three microliters of bacterial suspension (OD_600_ of 1.0) were dropped onto the motility plates which were incubated at 28 °C for 7 days. The diameter of colony expansion was measured. The assay was repeated at least three times with five biological replicates.

### 4.6. EPS Production and Biofilm Formation 

EPS production was determined as described previously [38]. To obtain maximum EPS, *Xoo* strains were adjusted to an OD_600_ of 0.3, serially diluted (1000-fold), and grown in 5 mL PSB for 5 days. One milliliter of bacterial cultures was centrifuged for 3 min at 10,500× *g* and 400 µL of the supernatant was mixed in 1.2 mL of 95% ethanol. After storing at −20°C for 1 day, the stored samples were centrifuged for 10 min at 16,500× *g* at 4 °C, desiccated for 1 hour, and dissolved in 1 mL of ultrapure water. One hundred microliters of the samples were added to 900 µL water, 1 mL of 5% aqueous phenol and 5 mL of H_2_SO_4_ in cold conditions. Mixed samples were measured at 488 nm by a spectrophotometer. Mean values from five biological replicates were determined and this experiment was repeated at least four times. Biofilm formation was measured by the polyvinyl chloride (PVC) microplate method as reported previously [37]. *Xoo* cells were incubated in PSB and adjusted to an OD_600_ of 0.3. One hundred microliters of bacterial suspensions were resuspended in 10 mL of XOM2 and incubated in PVC plates containing XOM2 media at 28 °C for 5 days. After eliminating supernatants, the remaining cells were stained using 0.1% crystal violet, washed two times with water, and dissolved in 95% ethanol. The dissolved samples were measured at 590 nm by a Spectramax 190 microplate reader (Molecular devices, Sunnyvale, CA, USA). This experiment was repeated six times with twelve biological replicates.

### 4.7. Siderophore Production

The chromeazurol S (CAS) assay was used to examine siderophore production as described previously [38]. PSA-CAS plates were used as the iron-rich medium. For the limited iron condition, 2,2′-dipyridyl was added in the PSA-CAS plates (PSA-CAS-BP). *Xoo* strains were incubated in PSA, harvested, washed, and adjusted to an OD_600_ of 0.6. Three microliters of bacterial suspension were dropped onto the plates. The halo zones were measured on plates after incubation for 7 days at 28 °C. This experiment was repeated six times with four biological replicates.

### 4.8. Statistical Analyses

All statistical analyses were accomplished using SPSS 12.0K (Statistical Package for the Social Sciences; SPSS Inc., Chicago, IL, USA). ANOVA and Tukey’s multiple comparison were used to determine significant difference (*p* < 0.05).

## Supplementary Materials

Supplementary materials can be found at http://www.mdpi.com/1422-0067/19/10/3038/s1.
