# Supplementary material for: Elucidating Functions of FleQ in Xanthomonas oryzae pv. oryzae by Comparative Proteomic and Phenotypic Analyses
Source: Int J Mol Sci. 2018 Oct 5;19(10):3038. doi: 10.3390/ijms19103038 (PMC6213323; doi:10.3390/ijms19103038)
Supplement: Supplementary file 1 [file ijms-19-03038-s001.zip › Figure S1.pdf]

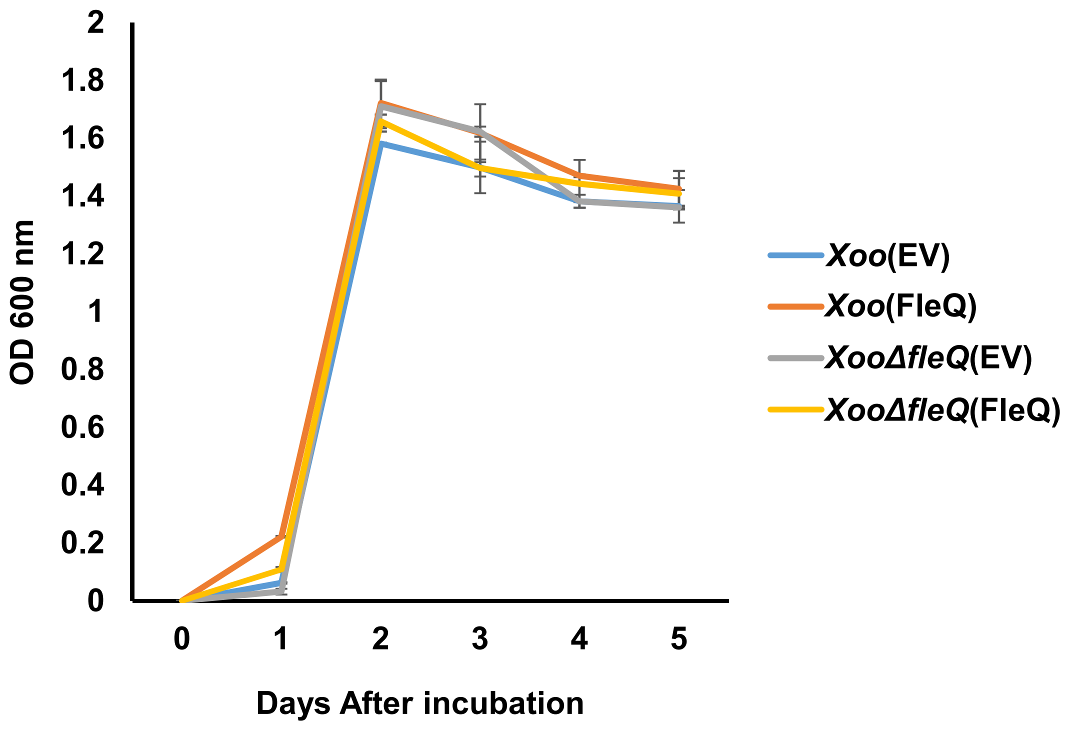

Figure S1. Growth of *Xoo*(EV), *Xoo*(FleQ), *XooΔfleQ*(EV) and *XooΔfleQ*(FleQ) in PSB media. *Xoo* strains were grown, adjusted OD_{600} of 0.3, serially diluted (10^{-3}), and grown in PSB media. Values of optical density (OD) at 600 nm were measured at 0, 1, 2, 3, 4, and 5 days after incubation. Data points indicate the means of three biological replicates with the standard deviations.
