# Supplementary material for: Elucidating Functions of FleQ in Xanthomonas oryzae pv. oryzae by Comparative Proteomic and Phenotypic Analyses
Source: Int J Mol Sci. 2018 Oct 5;19(10):3038. doi: 10.3390/ijms19103038 (PMC6213323; doi:10.3390/ijms19103038)
Supplement: Supplementary file 1 [file ijms-19-03038-s001.zip › Table S1_revised.pdf]

Table S1. Numbers of proteins and PSM (peptide spectral matches) detected in three biological replicates of *Xoo*(EV) and *Xoo*(FleQ) from LC-MS/MS.

Strains	1st		2nd		3rd		shared proteins in 3 biological replicates
	protein	PSM	protein	PSM	protein	PSM	
Xoo(EV)*	1407	80137	1442	79940	1416	80069	1291
Xoo(FleQ)	1405	77960	1447	78010	1367	78154	1244

*Identified proteins and PSMs from *Xoo*(EV) were used in a study published recently [33].
