# Supplementary material for: Elucidating Functions of FleQ in Xanthomonas oryzae pv. oryzae by Comparative Proteomic and Phenotypic Analyses
Source: Int J Mol Sci. 2018 Oct 5;19(10):3038. doi: 10.3390/ijms19103038 (PMC6213323; doi:10.3390/ijms19103038)
Supplement: Supplementary file 1 [file ijms-19-03038-s001.zip › Table S2_revised.pdf]

Table S2. Numbers of proteins and PSM (peptide spectral matches) detected in three biological replicates of *Xoo* and *XooΔfleQ* from LC-MS/MS.

Strains	1st		2nd		3rd		shared proteins in 3 biological replicates
	protein	PSM	protein	PSM	protein	PSM	
*Xoo**	1584	39360	1702	39605	2642	31602	1227
XooΔfleQ	1796	29283	1799	29285	2090	29243	1161

*Identified proteins and PSMs from *Xoo* were used in a study published recently [33].
