# Supplementary material for: Elucidating Functions of FleQ in Xanthomonas oryzae pv. oryzae by Comparative Proteomic and Phenotypic Analyses
Source: Int J Mol Sci. 2018 Oct 5;19(10):3038. doi: 10.3390/ijms19103038 (PMC6213323; doi:10.3390/ijms19103038)
Supplement: Supplementary file 1 [file ijms-19-03038-s001.zip › Table S3_revised.pdf]

Table S3. Classification of less abundant expressed (>2 fold) protein in *Xoo* (FleQ) using clusters of orthologous groups from the comparison between *Xoo* (EV) and *Xoo* (FleQ)

COG function	Accession	locus taq	Predicted function	fold change [*Xoo* (FleQ)/Xoo(EV)]	P-value	included in other group
C (Energy production and conversion)	188576165	PXO_00406	acetoacetyl-CoA reductase	0.36	0.038	
	188577544	PXO_01440	ketoglutarate semialdehyde dehydrogenase	0.41	0.002	
	188578691	PXO_03130	gloA lactoylglutathione lyase	*		
E (Amino acid transport and metabolism)	188574714	PXO_03755	5-methyltetrahydropteroyltriglutamate- homocysteine methyltransferase	0.42	0.024	
	188574895	PXO_04017	bifunctional PutA protein	0.43	0.001	
	188575454	PXO_04559	asparaginase	0.46	0.011	
	188575529	PXO_04482	gcvP glycine dehydrogenase	0.49	0.001	
	188576104	PXO_00463	glutamine synthetase	0.32	0.010	
	188577316	PXO_01684	sdaA L-serine ammonia-lyase	0.28	0.011	
	188577545	PXO_01439	dihydrodipicolinate synthetase	0.26	0.000	
	188576965	PXO_06174	nucleotide sugar transaminase	*		
	188577543	PXO_01441	proline dipeptidase	*		
	188578147	PXO_02190	synthetase/amidase	*		
F (Nucleotide metabolism	188578255	PXO_02361	inosine-uridine preferring nucleoside hydrolase	0.32	0.022	
	188575147	PXO_04267	cytosine deaminase	*		
G (Carbohydrate transport and metabolism)	188574381	PXO_03375	glgB 1,4-alpha-glucan branching enzyme	0.37	0.027	
	188574382	PXO_03374	treZ malto-oligosyltrehalose trehalohydrolase	0.27	0.024	
	188574384	PXO_03372	treY malto-oligosyltrehalose synthase	0.42	0.028	
	188574462	PXO_03668	alpha-amylase family protein	0.42	0.000	
	188574463	PXO_03667	alpha-amylase	0.38	0.007	
	188575952	PXO_00121	galactonate dehydratase	0.43	0.008	
	188578741	PXO_03080	L-sorbosone dehydrogenase	0.40	0.010	
	188577523	PXO_01470	hypothetical protein	*		
	188578082	PXO_02260	alpha-N-arabinofuranosidase 2	*		
	188578931	PXO_02887	glycosyl hydrolase, family 15	*		
H (Coenzyme transport and metabolism)	188575173	PXO_04293	nicotinamide phosphoribosyltransferase	0.39	0.003	
	188575168	PXO_04288	lipB lipoyltransferase	*		
	188578049	PXO_02141	adenosyl cobinamide kinase; adenosyl cobinamide phosphate guanylyltransferase	*		
I (Lipid metabolism)	188574549	PXO_03577	cardiolipin synthase	0.27	0.008	
	188574667	PXO_03805	acyl-CoA dehydrogenase	0.48	0.011	
	188575762	PXO_05532	enoyl-CoA hydratase/isomerase family protein	0.20	0.000	
	188578563	PXO_02706	3-oxoacyl-[acyl-carrier-protein] synthase III	0.43	0.000	
	188575774	PXO_05537	ispH 4-hydroxy-3-methylbut-2-enyl diphosphate reductase	*		
	188576967	PXO_06176	3-oxoacyl-ACP synthase	*		
	188577189	PXO_01811	PHB depolymerase	*		
J (Translation)	188575692	PXO_04827	ribosomal large subunit pseudouridine synthase F	*		
	188578072	PXO_02148	16S rRNA-processing protein RimM	*		
	188578438	PXO_02549	glu-trnAgln amidotransferase a subunit	*		
K (Transcription)	188574628	PXO_03849	sal operon transcriptional repressor	0.31	0.005	
	188575426	PXO_04591	oxidative stress transcriptional regulator	*		
	188577059	PXO_01234	ArsR family transcriptional regulator	*		
	188577334	PXO_01665	transcriptional regulator	*		
	188578021	PXO_01953	HrpX/HrpB	*		
L (Replication, recombination and repair)	188577111	PXO_01182	recN DNA repair protein RecN	0.46	0.008	
	188578568	PXO_02701	cas crispr-associated helicase Cas3 domain protein	0.31	0.000	
	188578573	PXO_02696	cas crispr-associated protein Cas1	0.32	0.018	
	188574426	PXO_03333	ATP-dependent RNA helicase	*		
	188574575	PXO_05437	exodeoxyribonuclease V subunit gamma	*		
	188575308	PXO_04356	dgtp-pyrophosphohydrolase; thiamine phosphate synthase	*		
	188575552	PXO_04456	-3-methyladenine glycosylase	*		
	188576896	PXO_06102	putative transposase	*		
M (Cell wall/membrane /envelope biogenesis)	188574745	PXO_03724	pathogenicity protein	0.40	0.033	
	188575376	PXO_04642	organic solvent tolerance protein	0.43	0.000	
	188575564	PXO_04696	imcF ImcF-related family	0.48	0.010	
	188576974	PXO_06183	O-antigen biosynthesis protein	0.05	0.014	
	188577399	PXO_01594	glycosyltransferase	0.29	0.011	
	188574338	PXO_03413	XopF1 effector	*		
	188575265	PXO_04400	hypothetical protein	*		
	188575823	PXO_00250	SciS protein	*		
	188576508	PXO_00587	lpxK tetraacyldisaccharide 4'-kinase	*		
	188577579	PXO_01403	exopolysaccharide xanthan biosynthesis glycosyltransferase GumM	*		
	188577582	PXO_01400	exopolysaccharide xanthan biosynthesis protein GumJ	*		
	188577583	PXO_01399	exopolysaccharide xanthan biosynthesis glycosyltransferase GumI	*		
	188577584	PXO_01398	exopolysaccharide xanthan biosynthesis glycosyltransferase GumH	*		
	188577589	PXO_01394	exopolysaccharide xanthan biosynthesis glycosyltransferase GumD	*		
	188577591	PXO_01391	exopolysaccharide xanthan biosynthesis export protein GumB	*		
	188578508	PXO_02622	3-deoxy-D-manno-octulosonic-acid transferase	*		
N (Cell motility)	188577157	PXO_01137	pili assembly chaperone	*		U
O (Post-translational modification, protein turnover, and	188575448	PXO_04567	domain of unknown function protein	0.20	0.015	
	188576457	PXO_00635	peptidase S8 and S53, subtilisin, kexin, sedolisin	0.17	0.038	
	188577071	PXO_01222	ABC transporter vitamin B12 uptake permease	0.11	0.002	
	188577953	PXO_02027	pirin	0.42	0.008	

turnover, and chaperones)	188578601	PXO_02667	disulfide isomerase	*		
P (Inorganic ion transport and metabolism)	188574287	PXO_03467	TonB-dependent outer membrane Receptor	0.20	0.001	
	188574650	PXO_03827	TonB-dependent receptor	0.31	0.000	
	188574892	PXO_04013	bfr bacterioferritin	0.34	0.036	
	188575259	PXO_04406	TonB-dependent outer membrane Receptor	0.30	0.001	
	188578215	PXO_02318	modA molybdate ABC transporter, periplasmic molybdate-binding protein	0.36	0.020	
	188578387	PXO_02495	ppk polyphosphate kinase	0.31	0.001	
	188578540	PXO_02728	TonB-dependent outer membrane receptor	0.31	0.016	
	188578988	PXO_02830	catalase	0.46	0.000	
	188579012	PXO_02805	TonB-dependent receptor	0.39	0.000	
	188576115	PXO_00453	magnesium and cobalt transport protein	*		
Q (Secondary metabolites biosynthesis, transport, and catabolism)	188575037	PXO_04215	hmgA homogentisate 1,2-dioxygenase	0.43	0.011	
	188574829	PXO_03950	AMP-ligase	*		
	188577188	PXO_01812	hypothetical protein	*		
	188577963	PXO_02015	dienelactone hydrolase	*		
	188578635	PXO_03182	ABC transporter ATP-binding protein	*		
S (Function unknown)	188575560	PXO_04448	hypothetical protein	0.18	0.006	
	188577462	PXO_01531	Rhs element Vgr protein	0.45	0.010	
	188578584	PXO_02685	general secretion pathway protein N	0.44	0.007	
	188574321	PXO_03429	putative secreted protein	*		
	188574341	PXO_03410	HrpD6	*		
	188574402	PXO_03356	hypothetical protein, XopW	*		
	188574484	PXO_03644	Rhs element Vgr protein	*		
	188574548	PXO_03578	yceI YceI like family	*		
	188574925	PXO_04047	hypothetical protein	*		
	188575572	PXO_05513	Sulfatase modifying factor 1 -like protein (C-alpha-formyglycine-generating enzyme 1)	*		
	188576534	PXO_00563	helix-destabilizing protein	*		
	188577418	PXO_01574	hypothetical protein	*		
	188577901	PXO_02079	hypothetical protein	*		
	188577923	PXO_02056	hypothetical protein	*		
	188577947	PXO_02033	hypothetical protein	*		
	188577951	PXO_02029	Rhs element Vgr protein	*		
	188578244	PXO_02349	transposase	*		
	188578350	PXO_02458	hypothetical protein	*		
	188578351	PXO_02459	hypothetical protein	*		
	188578431	PXO_02542	ISXo8 transposase	*		
	188574363	PXO_03390	hypothetical protein	0.40	0.009	
	188574397	PXO_03361	VirK	0.36	0.001	
	188574624	PXO_03853	sialic acid-specific 9-O-acetylesterase	0.33	0.045	
	188575286	PXO_04379	LppC superfamily	0.31	0.001	
	188575394	PXO_04623	hypothetical protein	0.06	0.000	
	188575565	PXO_04697	hypothetical protein	0.42	0.002	
	188575567	PXO_04699	impA ImpA, N-terminal	0.34	0.049	
	188575570	PXO_04702	lipoprotein, putative	0.29	0.000	
	188575752	PXO_04863	hypothetical protein	0.22	0.031	
	188575758	PXO_04857	hypothetical protein	0.40	0.002	
	188576006	PXO_00059	beta-ketoadipate enol-lactone hydrolase	0.39	0.016	
	188576411	PXO_00684	alkaline phosphatase	0.40	0.002	
	188576618	PXO_00916	methyl-accepting chemotaxis protein	0.48	0.040	
	188577066	PXO_01227	hypothetical protein	0.46	0.035	
	188577219	PXO_01779	dehydrogenase	0.36	0.048	
	188577580	PXO_01402	gumL exopolysaccharide xanthan biosynthesis pyruvyltransferase GumL	0.17	0.000	
	188577930	PXO_02049	EvpF	0.38	0.008	
	188577970	PXO_02005	rickettsia 17 kDa surface antigen family	0.48	0.048	
	188578139	PXO_02202	tryptophan halogenase	0.36	0.029	
	188579014	PXO_02803	pass1 domain protein	0.47	0.015	
	188579015	PXO_02802	tryptophan halogenase	0.47	0.008	
	188579056	PXO_02760	XopN effector	0.29	0.000	
	188579071	PXO_02746	polyvinylalcohol dehydrogenase	0.23	0.010	
	188579212	PXO_03539	putative ATP-binding protein	0.26	0.004	
	188579216	PXO_03536	hypothetical protein	0.48	0.004	
	188574334	PXO_03417	HrpF	*		
	188574337	PXO_03414	Hpa3	*		
	188574350	PXO_03401	type III secretion protein HrpB1/HrpK	*		
	188574581	PXO_03901	XopQ effector	*		
	188574618	PXO_03859	hypothetical protein	*		
	188575158	PXO_04253	Rhs element Vgr protein	*		
	188575324	PXO_04340	sulfotransferase	*		
	188575334	PXO_04687	phosphoanhydride phosphohydrolase	*		
	188575393	PXO_04624	hypothetical protein	*		
	188575458	PXO_04555	tRNA-processing ribonuclease BN	*		
	188575846	PXO_00227	pthXo1 TAL effector AvrBs3/PthA	*		
	188576022	PXO_00043	chemotaxis protein	*		
	188576163	PXO_00408	GumN protein	*		
	188576869	PXO_06072	hypothetical protein	*		
	188576973	PXO_06182	fkbM methyltransferase, FkbM family protein	*		
	188577156	PXO_01138	protein U	*		

	188577370	PXO_01625	hypothetical protein, XopK	*		
	188577374	PXO_01620	leucin rich protein, XopL	*		
	188577650	PXO_01334	hypothetical protein	*		
	188577707	PXO_01277	hypothetical protein	*		
	188577726	PXO_01258	SCO1/SenC superfamily	*		
	188577775	PXO_01885	outer membrane protein	*		
	188578140	PXO_02201	pass1 domain-containing protein	*		
	188578277	PXO_02383	hypothetical protein	*		
	188578355	PXO_02463	hypothetical protein	*		
	188578964	PXO_02855	metallo-beta-lactamase superfamily protein	*		
	188579013	PXO_02804	protein SapC protein	*		
T (Signal transduction mechanisms)	188574844	PXO_03965	two-component system regulatory protein	0.38	0.018	
	188574378	PXO_03378	signal protein with GGDEF domain	*		
	188574792	PXO_03912	anti-sigma F factor antagonist	*		
	188575701	PXO_04836	two-component system regulatory protein	*		
	188576990	PXO_06199	signal protein with GGDEF domain	*		
	188578299	PXO_02406	two-component system sensor protein	*		
	188578360	PXO_02467	protein phosphatase	*		
U (Intracellular trafficking,	188575475	PXO_04537	secE preprotein translocase subunit SecE	*		
	188578598	PXO_02671	xadA Xanthomonas adhesin-like protein A	*		W
V (Defense mechanisms)	188574417	PXO_03342	ABC transporter ATP-binding protein	0.28	0.002	
	188577915	PXO_02064	type I restriction enzyme EcoKI M protein	0.46	0.027	
	188576236	PXO_00334	cydD ABC transporter, CydDC cysteine exporter (CydDC-E) family, permease/ATP-binding protein CydD	*		
	188576249	PXO_00322	RND superfamily protein	*		
	188578532	PXO_02735	hypothetical protein	*		

* indicates the protein is unique to the *Xoo* (EV)
