# Supplementary material for: Elucidating Functions of FleQ in Xanthomonas oryzae pv. oryzae by Comparative Proteomic and Phenotypic Analyses
Source: Int J Mol Sci. 2018 Oct 5;19(10):3038. doi: 10.3390/ijms19103038 (PMC6213323; doi:10.3390/ijms19103038)
Supplement: Supplementary file 1 [file ijms-19-03038-s001.zip › Table S4_revised.pdf]

Table S4. Classification of more abundant (>2 fold) protein in *Xoo* (FleQ) using clusters of orthologous groups from the comparison between *Xoo* (EV) and *Xoo* (FleQ)

COG function	Accession	locus taq	predicted function	fold change [Xoo(FleQ)/ Xoo(EV)]	P-value	included in other group
C (Energy production and conversion)	188577235	PXO_01763	GTN Reductase	2.48	0.00019	
	188577683	PXO_01300	NADH-ubiquinone oxidoreductase Nqo14 subunit	*		
	188577689	PXO_01294	NADH-ubiquinone oxidoreductase chain 1	*		
	188577723	PXO_01261	glyoxylate reductase	*		
E (Amino acid transport and metabolism)	188576221	PXO_00350	acetylornithine deacetylase	4.32	0.00380	
	188576366	PXO_00728	hutG N-formylglutamate amidohydrolase	2.27	0.04312	
	188576575	PXO_00815	homoserine kinase	4.06	0.02208	
	188577148	PXO_01146	asnB asparagine synthase (glutamine-hydrolyzing)	2.94	0.01004	
F (Nucleotide metabolism and transport)	188576584	PXO_00808	pyrD dihydroorotate oxidase	*		
	188577983	PXO_01992	hypothetical protein	*		
	188578897	PXO_02921	deoxyuridine 5'-triphosphate nucleotidohydrolase	*		
G (Carbohydrate transport and metabolism)	188574991	PXO_04111	dehydrogenase	*		
	188575395	PXO_04622	gluconolactonase	*		
	188576074	PXO_00488	1-phosphofructokinase	*		
	188576653	PXO_00880	sugar ABC transporter substrate-binding protein	*		
	188577064	PXO_01229	glucose-galactose transporter	*		
	188577803	PXO_01859	glycogen debranching protein GlgX	*		
	188578121	PXO_02220	phosphotransferase system HPr enzyme	*		
H (Coenzyme transport and metabolism)	188576405	PXO_00690	panB 3-methyl-2-oxobutanoate hydroxymethyltransferase	2.62	0.00420	
	188578488	PXO_02603	thiC thiamine biosynthesis protein ThiC	4.06	0.04374	
	188575466	PXO_04547	hemA glutamyl-tRNA reductase	*		
	188576886	PXO_06090	molybdopterin biosynthesis protein MoeB	*		
I (Lipid metabolism)	188577058	PXO_01235	acyl-CoA dehydrogenase, short-chain specific	2.64	0.02269	
	188574498	PXO_03629	oxidoreductase	*		
	188574551	PXO_03575	acetyltransferase	*		
	188577167	PXO_01127	undecaprenyl pyrophosphate synthase	*		
J (Translation)	188576927	PXO_06136	infA translation initiation factor IF-1	2.82	0.03306	
	188575503	PXO_04508	30S ribosomal protein S14	*		
	188576349	PXO_00745	30S ribosomal protein S18	*		
	188579180	PXO_03239	50S ribosomal protein L33	*		
K (Transcription)	188576964	PXO_06173	FleQ	55.73	0.00000	
	188577025	PXO_06237	transcriptional regulator	2.06	0.00772	
	188575636	PXO_04819	Fur family transcriptional regulator	*		
L (Replication, recombination and repair)	188574271	PXO_03483	dnaA chromosomal replication initiator protein DnaA	2.82	0.03046	
	188574693	PXO_03776	deoxyribonuclease TatD	*		
	188576332	PXO_00762	exonuclease I	*		
	188576930	PXO_06139	7 and8-dihydro-8-oxoguanine-triphosphatase	*		
	188577602	PXO_01379	hypothetical protein	*		
	188577870	PXO_02112	isrso5-transposase	*		
M (Cell wall/membrane/ envelope biogenesis)	188577138	PXO_01155	outer membrane efflux protein	2.45	0.00692	
	188578233	PXO_02338	ErfK/YbiS/YcfS/YnhG	2.76	0.01417	
	188574945	PXO_04067	lipid A biosynthesis lauroyl acyltransferase	*		
	188575702	PXO_04837	outer membrane channel protein	*		
	188577965	PXO_02013	UDP-N-acetylmuramoyl-L-alanyl-D-glutamate synthetase	*		
	188578344	PXO_02452	heptosyl transferase	*		
N (Cell motility)	188576955	PXO_06164	flagellar hook-associated protein FlgL	*		
	188576981	PXO_06190	flagellar protein	*		
O (Post-translational modification, protein turnover, and chaperones)	188577996	PXO_01979	sufB FeS assembly protein SufB	2.33	0.02183	
	188576437	PXO_00658	glutathione peroxidase	*		
	188577998	PXO_01977	FeS assembly protein SufD	*		
P (Inorganic ion transport and metabolism)	188577853	PXO_02129	TonB-dependent receptor	2.47	0.01730	
	188575279	PXO_04386	potassium-transporting ATPase subunit B	*		
	188576272	PXO_00303	TonB-dependent outer membrane receptor	*		
	188577531	PXO_01457	TonB-dependent outer membrane receptor	*		
	188578151	PXO_02186	voltage-gated ion channel superfamily protein	*		
	188578406	PXO_02512	TonB-dependent receptor	*		
Q (Secondary metabolites biosynthesis, transport, and catabolism)	188579087	PXO_03209	toluene tolerance protein	2.04	0.01182	
	188578910	PXO_02909	3-oxoadipate enol-lactonase	*		
	188575364	PXO_04653	lemA LemA family protein	2.86	0.01712	
	188576204	PXO_00366	general stress protein	3.01	0.02667	
	188576230	PXO_00338	alpha-amino acid ester hydrolase	2.50	0.00727	
	188574318	PXO_03432	ISXoo2 transposase	2.04	0.04766	
	188575255	PXO_04411	hypothetical protein	2.21	0.04065	
	188574393	PXO_03365	ISXo8 transposase	*		
	188574582	PXO_03899	lipoprotein	*		

	188574607	PXO_03873	hypothetical protein	*		
	188575575	PXO_04718	lipoprotein	*		
	188576458	PXO_00634	ISXoo8 transposase	*		
	188577119	PXO_01174	lipoprotein	*		
	188577493	PXO_01501	ISXoo6 transposase	*		
	188577949	PXO_02031	hypothetical protein	*		
	188578567	PXO_02702	ISXo5 transposase	*		
	188579089	PXO_03207	hypothetical protein	*		
	188579095	PXO_03202	ISXo2 transposase	*		
	188576372	PXO_00722	poly(hydroxyalcanoate) granule associated protein	5.42	0.04223	
	188577530	PXO_01458	hypothetical protein	3.59	0.00790	
S (Function unknown)	188577987	PXO_01988	bacterial DNA-binding protein	2.86	0.00302	
	188579206	PXO_03545	hypothetical protein	4.11	0.00298	
	188574374	PXO_03381	ABC transporter substrate binding protein	*		
	188574439	PXO_03320	hypothetical protein	*		
	188574515	PXO_03613	tryptophan halogenase	*		
	188574837	PXO_03958	lipoprotein	*		
	188574881	PXO_04002	nitrilotriacetate monooxygenase component B	*		
	188574928	PXO_04050	RDD family protein	*		
	188574941	PXO_04063	membrane flanked domain family	*		
	188575569	PXO_04701	DNA repair ATPase	*		
	188576461	PXO_00631	cointegrate resolution protein T	*		
	188576621	PXO_00911	inner membrane protein YeiH	*		
	188576933	PXO_06142	chemotaxis protein	*		
	188577429	PXO_01563	protein TolA	*		
	188577941	PXO_02039	EF hand domain-containing protein	*		
	188578105	PXO_02236	hypothetical protein	*		
	188578381	PXO_02489	carboxymuconolactone decarboxylase	*		
	188578512	PXO_02626	hypothetical protein	*		
	188579028	PXO_02789	hypothetical protein	*		
	188579254	PXO_03489	tRNA modification GTPase TrmE	*		
T (Signal transduction mechanisms)	188575610	PXO_04751	chemotaxis protein	*		
	188575915	PXO_00155	RNA chaperone Hfq	*		
	188576596	PXO_00798	two-component system sensor protein	*		
	188577309	PXO_01691	histidine kinase/response regulator hybrid protein	*		
	188577310	PXO_01690	histidine kinase-response regulator hybrid protein	*		
U (Intracellular trafficking, secretion, and vesicular transport)	188576290	PXO_00521	secF protein-export membrane protein SecF	3.13	0.00098	
V (Defense mechanisms)	188577137	PXO_01156	multidrug resistance efflux pump	4.26	0.00037	
	188575881	PXO_00189	ABC transporter ATP-binding protein	*		

* indicates the protein is unique to the Xoo(FleQ)
