# Supplementary material for: Elucidating Functions of FleQ in Xanthomonas oryzae pv. oryzae by Comparative Proteomic and Phenotypic Analyses
Source: Int J Mol Sci. 2018 Oct 5;19(10):3038. doi: 10.3390/ijms19103038 (PMC6213323; doi:10.3390/ijms19103038)
Supplement: Supplementary file 1 [file ijms-19-03038-s001.zip › Table S5_revised.pdf]

**Table S5. Classification of less abundant (>2 fold) protein in *XooΔfleQ* using clusters of orthologous groups from the comparison between *XooΔfleQ* and *Xoo***

| COG function | Accession | locus taq | Predicted function | fold change (*XooΔfleQ* /*Xoo*) | P-value | included in other group |
|---|---|---|---|---|---|---|
| C (Energy production and conversion) | 188574291 | PXO_03463 | L-lactate dehydrogenase | 0.50 | 0.0043 | |
| | 188577685 | PXO_01298 | NADH-quinone oxidoreductase chain I | 0.47 | 0.0040 | |
| | 188578714 | PXO_03110 | atpH ATP synthase F1 and delta subunit | 0.48 | 0.0014 | |
| | 188574429 | PXO_03330 | avirulence protein AvrBs2 | * | | |
| | 188574660 | PXO_03815 | cytochrome C5 | * | | |
| | 188575437 | PXO_04578 | nitroreductase family | * | | |
| | 188576377 | PXO_00718 | twin-arginine translocation pathway signal | * | | |
| | 188577687 | PXO_01296 | NADH-ubiquinone oxidoreductase Nqo10 subunit | * | | |
| | 188577758 | PXO_01901 | glutamate symport protein | * | | |
| | 188578533 | PXO_02734 | glycerophosphodiester phosphodiesterase | * | | |
| | 188578925 | PXO_02893 | acetyltransferase, gnat family | * | | K |
| E (Amino acid transport and metabolism) | 188574441 | PXO_03319 | glutamate synthase | 0.44 | 0.0044 | |
| | 188575123 | PXO_04132 | prolyl oligopeptidase family protein | 0.46 | 0.0086 | |
| | 188575946 | PXO_00126 | dapA dihydrodipicolinate synthase | 0.45 | 0.0004 | |
| | 188576104 | PXO_00463 | glutamine synthetase | 0.36 | 0.0108 | |
| | 188576283 | PXO_00528 | L-lysine 6-aminotransferase | 0.42 | 0.0173 | |
| | 188578413 | PXO_02519 | kbl 2-amino-3-ketobutyrate coenzyme A ligase | 0.33 | 0.0008 | |
| | 188574714 | PXO_03755 | 5-methyltetrahydropteroyltriglutamate- homocysteine methyltransferase | * | | |
| | 188575357 | PXO_04660 | acyl-CoA thioesterase I | * | | |
| | 188575891 | PXO_00179 | serine carboxypeptidase | * | | |
| | 188576920 | PXO_06127 | hypothetical protein | * | | |
| | 188576965 | PXO_06174 | nucleotide sugar transaminase | * | | |
| | 188577042 | PXO_06257 | metX homoserine O-acetyltransferase | * | | |
| | 188577253 | PXO_01746 | leucine dehydrogenase | * | | |
| | 188577712 | PXO_01272 | trpA tryptophan synthase, alpha subunit | * | | |
| F (Nucleotide metabolism and transport) | 188578397 | PXO_02504 | purF amidophosphoribosyltransferase | 0.45 | 0.0097 | |
| | 188575371 | PXO_04647 | bis(5'-nucleosyl)-tetraphosphatase (symmetrical) | * | | |
| | 188576902 | PXO_06108 | GTP-binding protein | * | | |
| | 188578974 | PXO_02846 | tmk thymidylate kinase | * | | |
| G (Carbohydrate transport and metabolism) | 188574285 | PXO_03469 | fbp fructose-1 and6-bisphosphatase | 0.49 | 0.0011 | |
| | 188578278 | PXO_02384 | rpiA ribose 5-phosphate isomerase A | 0.50 | 0.0328 | |
| | 188578403 | PXO_02509 | phosphoglycerate mutase | 0.47 | 0.0063 | |
| | 188574298 | PXO_03455 | 2-keto-3-deoxygluconate kinase | * | | |
| | 188575748 | PXO_04867 | otsB trehalose-phosphatase | * | | |
| | 188577271 | PXO_01728 | beta-hexosaminidase | * | | |
| | 188577698 | PXO_01285 | tpiA triosephosphate isomerase | * | | |
| | 188578122 | PXO_02219 | ptsI phosphoenolpyruvate-protein phosphotransferase | * | | |
| H (Coenzyme transport and metabolism) | 188575142 | PXO_04274 | ubiE ubiquinone/menaquinone biosynthesis methyltransferase UbiE | 0.43 | 0.0024 | |
| | 188575172 | PXO_04292 | bifunctional NMN adenylyltransferase/Nudix hydrolase | 0.40 | 0.0119 | |
| | 188579029 | PXO_02788 | panE 2-dehydropantoate 2-reductase | 0.43 | 0.0149 | |
| | 188579047 | PXO_02769 | ubiB 2-polyprenylphenol 6-hydroxylase | 0.31 | 0.0099 | |
| | 188574497 | PXO_03630 | bioC biotin biosynthesis protein BioC | * | | |
| | 188574888 | PXO_04009 | ubiquinone biosynthesis protein | * | | |
| | 188575036 | PXO_04216 | hppD 4-hydroxyphenylpyruvate dioxygenase | * | | |
| | 188575239 | PXO_04425 | glutamate--cysteine ligase | * | | |
| | 188576405 | PXO_00690 | panB 3-methyl-2-oxobutanoate hydroxymethyltransferase | * | | |
| | 188577962 | PXO_02016 | ispb polyprenyl synthetase | * | | |
| | 188578154 | PXO_02183 | pyridoxamine 5'-phosphate oxidase | * | | |
| | 188578945 | PXO_02874 | bioB biotin synthase | * | | |
| | 188579217 | PXO_03535 | folE GTP cyclohydrolase I | * | | |
| I (Lipid metabolism) | 188574520 | PXO_03607 | glycerol-3-phosphate acyltransferase | 0.43 | 0.0015 | |
| | 188575774 | PXO_05537 | ispH 4-hydroxy-3-methylbut-2-enyl diphosphate reductase | 0.41 | 0.0295 | |
| | 188576594 | PXO_00800 | ispG 4-hydroxy-3-methylbut-2-en-1-yl diphosphate synthase | 0.43 | 0.0021 | |
| | 188577289 | PXO_01709 | fatty oxidation complex alpha subunit | 0.49 | 0.0153 | |
| | 188575391 | PXO_04626 | P-hydroxycinnamoyl CoA hydratase | * | | |
| | 188575902 | PXO_00168 | ispF 2C-methyl-D-erythritol 2,4-cyclodiphosphate synthase | * | | |
| | 188576967 | PXO_06176 | 3-oxoacyl- synthase | * | | |
| | 188577115 | PXO_01178 | streptomyces cyclase/dehydrase superfamily | * | | |
| | 188577167 | PXO_01127 | uppS undecaprenyl diphosphate synthase | * | | |
| | 188577293 | PXO_01706 | enoyl-CoA hydratase | * | | |
| | 188578040 | PXO_01931 | pvsA vibrioferrin biosynthesis protein PvsA | * | | |
| | 188578683 | PXO_03138 | fabA beta-hydroxyacyl-(acyl-carrier-protein) dehydratase FabA | * | | |
| | 188578914 | PXO_02905 | pcaF beta-ketoadipyl CoA thiolase | * | | |
| | 188574926 | PXO_04048 | Sua5/YciO/YrdC family protein | 0.42 | 0.0178 | |
| | 188575407 | PXO_04610 | glnS glutaminyl-tRNA synthetase | 0.48 | 0.0040 | |
| | 188575465 | PXO_04548 | prfA peptide chain release factor 1 | 0.33 | 0.0151 | |
| | 188576122 | PXO_00446 | miaB tRNA-I(6)A37 thiotransferase enzyme MiaB | 0.37 | 0.0089 | |

| | | | | | | |
|---|---|---|---|---|---|---|
| J (Translation) | 188577725 | PXO_01259 | hemK modification methylase and HemK family | 0.49 | 0.0374 | |
| | 188578564 | PXO_02705 | rpmF ribosomal protein L32 | 0.43 | 0.0008 | |
| | 188578611 | PXO_02657 | valS valyl-tRNA synthetase | 0.27 | 0.0024 | |
| | 188575498 | PXO_04513 | rpmC ribosomal protein L29 | * | | |
| | 188575910 | PXO_00160 | hypothetical protein | * | | |
| | 188575911 | PXO_00159 | rrmJ ribosomal RNA large subunit methyltransferase J | * | | |
| | 188576927 | PXO_06135 | infA translation initiation factor IF-1 | * | | |
| | 188577228 | PXO_01770 | fmtc oxacillin resistance-associated protein fmtc | * | | |
| K (Transcription) | 188575056 | PXO_04197 | N-acetyltransferase | * | | |
| | 188575920 | PXO_00150 | lexA LexA repressor | * | | |
| | 188575985 | PXO_00085 | transcriptional elongation factor | * | | |
| | 188576615 | PXO_00918 | iron dependent repressor | * | | |
| | 188576658 | PXO_00875 | morphogene BolA protein | * | | |
| | 188577059 | PXO_01234 | transcriptional regulator | * | | |
| | 188577216 | PXO_01782 | RNA polymerase sigma factor | * | | |
| | 188577282 | PXO_01717 | rnc ribonuclease III | * | | |
| L (Replication, recombination and repair) | 188574927 | PXO_04049 | topA DNA topoisomerase I | 0.31 | 0.0147 | |
| | 188575614 | PXO_04745 | mfd transcription-repair coupling factor | 0.45 | 0.0023 | |
| | 188575308 | PXO_04356 | dgtp-pyrophosphohydrolase; thiamine phosphate synthase | * | | |
| | 188576161 | PXO_00410 | mutL DNA mismatch repair protein MutL | * | | |
| | 188577414 | PXO_01578 | hrpA ATP-dependent helicase HrpA | * | | |
| | 188577974 | PXO_02001 | hypothetical protein | * | | |
| M (Cell wall/membrane /envelope biogenesis) | 188575294 | PXO_04370 | UDP-N-acetylmuramoyl-tripeptide--D-alanyl-D- alanine ligase | 0.23 | 0.0255 | |
| | 188578620 | PXO_02649 | soluble lytic murein transglycosylase | 0.39 | 0.0021 | |
| | 188574278 | PXO_03476 | TonB protein | * | | |
| | 188575153 | PXO_04259 | penicillin-binding protein 2 | * | | |
| | 188575781 | PXO_00004 | Rhs element Vgr protein, putative | * | | |
| | 188577221 | PXO_01777 | cyclopropane-fatty-acyl-phospholipid synthase | * | | |
| | 188577583 | PXO_01399 | gumI exopolysaccharide xanthan biosynthesis glycosyltransferase GumI | * | | |
| | 188578251 | PXO_02356 | penicillin-binding protein 1A (PBP-1a) (PBP1a) | * | | |
| N (Cell motility) | 188576036 | PXO_00027 | OmpA family protein,chemotaxis protein MotB | * | | |
| | 188576956 | PXO_06165 | flagellar protein | * | | |
| O (Post-translational modification, protein turnover, and chaperones) | 188577468 | PXO_01524 | gntY protein GntY | 0.47 | 0.0069 | |
| | 188575906 | PXO_00164 | pcm protein-L-isoaspartate O-methyltransferase | * | | |
| | 188577116 | PXO_01177 | smpB SsrA-binding protein | * | | |
| P (Inorganic ion transport and metabolism) | 188577777 | PXO_01883 | TonB-dependent outer membrane Receptor | 0.46 | 0.0100 | |
| | 188577853 | PXO_02129 | TonB-dependent receptor | 0.45 | 0.0126 | |
| | 188578039 | PXO_01932 | TonB-dependent outer membrane Receptor | 0.37 | 0.0148 | |
| | 188574966 | PXO_04088 | TonB-dependent outer membrane Receptor | * | | |
| | 188576273 | PXO_00302 | TonB-dependent receptor | * | | |
| | 188578214 | PXO_02317 | beta-lactamase | * | | |
| | 188579174 | PXO_03245 | alkaline phosphatase | * | | |
| Q (Secondary metabolites biosynthesis, transport, and catabolism) | 188578041 | PXO_01930 | pvsB vibrioferrin biosynthesis protein PvsB | * | | |
| | 188578635 | PXO_03182 | ABC transporter ATP-binding protein | * | | |
| | 188578689 | PXO_03132 | copA copper resistance protein A | * | | |
| | 188579087 | PXO_03209 | toluene tolerance protein | * | | |
| | 188574437 | PXO_03322 | lipoprotein, putative | 0.45 | 0.0224 | |
| | 188577927 | PXO_02052 | hypothetical protein | 0.43 | 0.0112 | |
| | 188574893 | PXO_04014 | ISXo5 transposase | * | | |
| | 188575111 | PXO_04142 | ISXoo13 transposase | * | | |
| | 188575568 | PXO_04700 | Rhs element Vgr protein | * | | |
| | 188575572 | PXO_05513 | Sulfatase modifying factor 1 precursor-like protein (C-alpha-formyglycine-generating enzyme 1) | * | | |
| | 188575784 | PXO_05538 | rhs RHS Repeat family | * | | |
| | 188575802 | PXO_00272 | hypothetical protein | * | | |
| | 188575878 | PXO_00193 | hypothetical protein | * | | |
| | 188576343 | PXO_00751 | hypothetical protein | * | | |
| | 188576458 | PXO_00634 | ISXoo8 transposase | * | | |
| | 188577477 | PXO_05663 | ISXoo12 transposase | * | | |
| | 188578617 | PXO_02738 | ISXo1 transposase | * | | |
| | 188579095 | PXO_03202 | ISXo2 putative transposase | * | | |
| | 188575565 | PXO_04697 | hypothetical protein | 0.48 | 0.0259 | |
| | 188576618 | PXO_00916 | methyl-accepting chemotaxis protein | 0.13 | 0.0034 | |
| | 188577094 | PXO_01199 | acetyltransferase and gnat family | 0.42 | 0.0175 | |
| | 188578392 | PXO_02499 | lpxH UDP-2 and3-diacylglucosamine hydrolase | 0.46 | 0.0230 | |
| | 188574374 | PXO_03381 | ABC transporter substrate binding protein | * | | |
| | 188574433 | PXO_03326 | hypothetical protein | * | | |
| | 188574475 | PXO_03654 | hypothetical protein | * | | |

| | | | | | | |
|---|---|---|---|---|---|---|
| S (Function unknown) | 188574484 | PXO_03644 | Rhs element Vgr protein | * | | |
| | 188574495 | PXO_03632 | hypothetical protein | * | | |
| | 188574513 | PXO_03615 | 5'-nucleotidase, lipoprotein e(P4) family | * | | |
| | 188574538 | PXO_03588 | hypothetical protein | * | | |
| | 188574572 | PXO_03554 | hemagglutinin-like protein | * | | |
| | 188574654 | PXO_03824 | ATPase | * | | |
| | 188574656 | PXO_03819 | hypothetical protein | * | | |
| | 188574813 | PXO_03933 | hypothetical protein | * | | |
| | 188574837 | PXO_03958 | lipoprotein, putative | * | | |
| | 188575178 | PXO_04298 | hypothetical protein | * | | |
| | 188575232 | PXO_04431 | GTP-binding protein | * | | |
| | 188575334 | PXO_04687 | phosphoanhydride phosphohydrolase | * | | |
| | 188575364 | PXO_04653 | lemA LemA family protein | * | | |
| | 188575536 | PXO_04472 | inner membrane protein | * | | |
| | 188575626 | PXO_04733 | hypothetical protein | * | | |
| | 188575757 | PXO_04858 | EF hand domain protein | * | | |
| | 188575967 | PXO_00103 | 3-hydroxyacyl-CoA dehydrogenase type II | * | | |
| | 188576022 | PXO_00043 | chemotaxis protein | * | | |
| | 188576647 | PXO_00886 | beta-alanine synthetase | * | | |
| | 188576966 | PXO_06175 | acyl carrier protein | * | | |
| | 188576973 | PXO_06182 | fkbM methyltransferase, FkbM family protein | * | | |
| | 188577156 | PXO_01138 | protein U | * | | |
| | 188577219 | PXO_01779 | dehydrogenase | * | | |
| | 188577682 | PXO_01301 | yhbC YhbC family protein | * | | |
| | 188577782 | PXO_01878 | domain of unknown function superfamily | * | | |
| | 188577946 | PXO_02034 | hypothetical protein | * | | |
| | 188577947 | PXO_02033 | hypothetical protein | * | | |
| | 188577957 | PXO_02024 | hydrolase | * | | |
| | 188578105 | PXO_02236 | hypothetical protein | * | | |
| | 188578111 | PXO_02230 | hypothetical protein | * | | |
| | 188578150 | PXO_02187 | hypothetical protein | * | | |
| | 188578313 | PXO_02422 | hypothetical protein | * | | |
| | 188578328 | PXO_02436 | methanol dehydrogenase regulator | * | | |
| | 188578355 | PXO_02463 | hypothetical protein | * | | |
| | 188578399 | PXO_02506 | hypothetical protein | * | | |
| | 188578950 | PXO_02868 | hydrolase | * | | |
| T (Signal transduction mechanisms) | 188574562 | PXO_03565 | two-component system sensor protein | * | | |
| | 188575184 | PXO_04304 | two-component system sensor protein | * | | |
| | 188575701 | PXO_04836 | two-component system regulatory protein | * | | |
| | 188576011 | PXO_00054 | methyl-accepting chemotaxis protein | * | | |
| | 188576018 | PXO_00047 | chemotaxis protein | * | | |
| | 188576019 | PXO_00046 | chemotaxis protein | * | | |
| | 188576021 | PXO_00045 | chemotaxis protein | * | | |
| | 188576023 | PXO_00041 | chemotaxis protein | * | | |
| | 188576031 | PXO_00032 | cheA chemotaxis protein CheA | * | | |
| | 188576936 | PXO_06145 | Putative signal protein with PAS(PAC), GGDEF and EAL domains | * | | |
| | 188577287 | PXO_01712 | regulatory protein | * | | |
| | 188578023 | PXO_01951 | hrpG HrpG | * | | |
| | 188578297 | PXO_02404 | transcriptional regulator LuxR/uhpA family | * | | |
| | 188578874 | PXO_02943 | two-component system sensor protein | * | | |
| U (Intracellular trafficking, secretion, and vesicular transport) | 188576289 | PXO_00522 | secD protein-export membrane protein SecD | 0.38 | 0.0019 | |
| | 188576287 | PXO_00524 | yajC preprotein translocase, YajC subunit | * | | |
| | 188577284 | PXO_01715 | lepB signal peptidase I | * | | |
| | 188577659 | PXO_01325 | type IV pilin | * | | |
| V (Defense mechanisms) | 188577137 | PXO_01156 | multidrug resistance efflux pump | * | | |
| | 188578532 | PXO_02735 | hypothetical protein | * | | |

* indicates that the protein is unique to *Xoo*
