# Supplementary material for: Elucidating Functions of FleQ in Xanthomonas oryzae pv. oryzae by Comparative Proteomic and Phenotypic Analyses
Source: Int J Mol Sci. 2018 Oct 5;19(10):3038. doi: 10.3390/ijms19103038 (PMC6213323; doi:10.3390/ijms19103038)
Supplement: Supplementary file 1 [file ijms-19-03038-s001.zip › Table S6_revised.pdf]

Table S6. Classification of more abundant (>2 fold) protein in *XooΔfleQ* using clusters of orthologous groups from the comparison between *XooΔfleQ* and *Xoo*

COG function	Accession	locus taq	Predicted function	fold change (*XooΔfleQ* /*Xoo*)	P-value	included in other group
E (Amino acid transport and metabolism)	188575980	PXO_00090	dapB dihydrodipicolinate reductase	2.16	0.003	
	188576562	PXO_00834	hisC histidinol-phosphate aminotransferase	4.16	0.046	
	188574879	PXO_04000	anthranilate synthase component II	*		
	188576564	PXO_00832	hisG ATP phosphoribosyltransferase	*		
	188578409	PXO_02515	cysA sulfate/thiosulfate import ATP-binding protein CysA	*		
F (Nucleotide	188576119	PXO_00449	hypothetical protein	2.14	0.009	
G (Carbohydrate transport and metabolism)	188574609	PXO_03869	beta-xylosidase (1,4-beta-D-xylan xylohydrolase)	*		
	188576327	PXO_00767	NAD(+) kinase	*		
	188578046	PXO_01925	alpha-ribazole-5''''-phosphate phosphatase	*		
	188578551	PXO_02718	prpB methylisocitrate lyase	*		
	188578931	PXO_02887	glycosyl hydrolase, family 15	*		
H (Coenzyme transport and metabolism)	188576261	PXO_00311	coaD pantetheine-phosphate adenylyltransferase	2.23	0.040	
	188575370	PXO_04648	folA dihydrofolate reductase	*		
	188577106	PXO_01187	pyridoxal kinase	*		
	188578400	PXO_02507	folylpolyglutamate synthase; dihydrofolate synthase	*		
I (Lipid	188575057	PXO_04196	hypothetical protein	*		
J (Translation)	188576349	PXO_00745	rpsR ribosomal protein S18	*		
	188579051	PXO_02765	peptidyl-tRNA hydrolase domain, putative	*		
L (Replication	188577280	PXO_01719	recO DNA repair protein RecO	*		
M (Cell wall/membrane/envelope biogenesis)	188575292	PXO_04372	ftsI peptidoglycan synthetase FtsI	*		
	188575715	PXO_04849	RhsD protein	*		
	188577176	PXO_01116	lpxB lipid-A-disaccharide synthase	*		
	188577398	PXO_01595	mrcB penicillin-binding protein 1B	*		
	188577965	PXO_02013	murD UDP-N-acetylmuramoylalanine--D-glutamate ligase	*		
	188578233	PXO_02338	ErfK/YbiS/YcfS/YnhG	*		
O (Post-translational	188574496	PXO_03631	aspartyl-asparaginyl beta-hydroxylase	*		
	188577953	PXO_02027	pirin	*		
P (Inorganic ion transport and metabolism)	188574611	PXO_03867	TonB-dependent receptor	7.07	0.002	
	188578143	PXO_02198	TonB-dependent receptor	5.53	0.003	
	188578988	PXO_02830	catalase	2.57	0.015	
	188575668	PXO_04790	TonB-dependent receptor	*		
	188578652	PXO_03166	wzt ABC transporter, ATP binding protein	*		
	188579175	PXO_03244	alkaline phosphatase D	*		
Q (Secondary	188577188	PXO_01812	hypothetical protein	*		
S (Function unknown)	188574360	PXO_03392	Hpa1	*		
	188575575	PXO_04718	lipoprotein, putative	*		
	188575717	PXO_04851	ISXo8 transposase	*		
	188576065	PXO_00498	hypothetical protein	*		
	188576202	PXO_00368	ISXo1 transposase	*		
	188576892	PXO_06098	hypothetical protein	*		
	188577949	PXO_02031	hypothetical protein	*		
	188578659	PXO_05753	hypothetical protein	*		
	188578677	PXO_03144	hypothetical protein	*		
	188579032	PXO_02785	putative ISXoo14 transposase	*		
	188575393	PXO_04624	hypothetical protein	*		
	188577368	PXO_01628	hypothetical protein	*		
	188577420	PXO_01572	ExsB protein	*		
	188577614	PXO_01368	hypothetical protein	*		
	188577940	PXO_02040	Rhs element Vgr protein	*		
	188578140	PXO_02201	pass1 domain protein	*		
	188578637	PXO_03180	lipoprotein, putative	*		
	188578774	PXO_03046	4,5-dopa dioxygenase extradiol	*		
T (Signal transduction mechanisms)	188576961	PXO_06170	response regulator	*		
U (Intracellular trafficking,	188577662	PXO_01322	prepilin-type N-terminal cleavage/methylation domain protein	*		
	188578598	PXO_02671	xadA Xanthomonas adhesin-like protein A	3.02	0.001	W
V (Defense mechanisms)	188577910	PXO_02070	type I restriction enzyme EcoAI R protein	*		

* indicates the protein is unique to the *XoofleQ*
