# Supplementary material for: Elucidating Functions of FleQ in Xanthomonas oryzae pv. oryzae by Comparative Proteomic and Phenotypic Analyses
Source: Int J Mol Sci. 2018 Oct 5;19(10):3038. doi: 10.3390/ijms19103038 (PMC6213323; doi:10.3390/ijms19103038)
Supplement: Supplementary file 1 [file ijms-19-03038-s001.zip › Table S7_revised.pdf]

Table S7. Bacterial strains and plasmids used in this study

Strains and plasmid	Characteristics	Source or reference
Escherichia coli		
DH5α	F-, `80*dlaczZΔM15,* Δ(*lacZY*A-argF), U169, *deoR*, RecA1, *end*A1, *hsd*R17, gyrA96, *thit*-1, r*el*A1, *supE44*	Promega
Xanthomonas oryzae pv. *oryzae*		
PXO99a	Wild type, Philippine race 6 strain; Cp^r	Hopkins et al., 1992
Xoo(EV)	Wild type strain carrying pBBR1-MCS5, Gm^r	This study
Xoo(FleQ)	Wild type strain carrying pBBR1FleQ, Gm^r	This study
XooΔfleQ(EV)	Insertional mutant in *fleQ* (*PXO_00993*) carrying pBBR1-MCS5, Km^r, Gm^r	This study
XooΔfleQ(FleQ)	*XooΔfleQ* complemented with pBBR1FleQ, Km^r, Gm^r	This study
Plasmid		
pGem-T-easy	TA cloning vector, Am^r	Promega
pGem-fleQ	pGEM-T easy inserted by 1603-bp *fleQ* fragment by TA ligation, Am^r	This study
pUC4K	Vector carrying kanamycin cassette, Km^r	Pharmacia
pGem-fleQ::km	A derivative of pGEM-fleQ. *fleQ* gene disrupted by kanamycine cassette, Am^r, Km^r	This study
pBBR1-MCS5	Broad-host-range vector, lac promoter, Gm^r	Kovach et al., 1995
pGemFleQ-OE	pGEM-T easy carrying a 1619-bp FleQ fragment containing 6xHis-tag at N-terminus at backward primer by TA ligation, Am^r	This study
pBBR1FleQ	pBBR1-MCS5 carrying a 1619-bp with 6xHis-tag at N-terminus from pGemFleQ-OE, Gm^r	This study

Cp^r, Am^r, Km^r and Gm^r represent resistance to cephalexin, ampicillin, kanamycin and gentamycin.
